# Genetic Evidence of Geographical Groups among Neanderthals

**DOI:** 10.1371/journal.pone.0005151

**Published:** 2009-04-15

**Authors:** Virginie Fabre, Silvana Condemi, Anna Degioanni

**Affiliations:** Laboratoire d'Anthropologie Bio-culturelle, UMR6578 Université de la Méditerranée -CNRS-EFS, Marseille, France; University of Montreal, Canada

## Abstract

The Neanderthals are a well-distinguished Middle Pleistocene population which inhabited a vast geographical area extending from Europe to western Asia and the Middle East. Since the 1950s paleoanthropological studies have suggested variability in this group. Different sub-groups have been identified in western Europe, in southern Europe and in the Middle East. On the other hand, since 1997, research has been published in paleogenetics, carried out on 15 mtDNA sequences from 12 Neanderthals. In this paper we used a new methodology derived from different bioinformatic models based on data from genetics, demography and paleoanthropology. The adequacy of each model was measured by comparisons between simulated results (obtained by BayesianSSC software) and those estimated from nucleotide sequences (obtained by DNAsp4 software). The conclusions of this study are consistent with existing paleoanthropological research and show that Neanderthals can be divided into at least three groups: one in western Europe, a second in the Southern area and a third in western Asia. Moreover, it seems from our results that the size of the Neanderthal population was not constant and that some migration occurred among the demes.

## Introduction

Neanderthals are a well-distinguished Middle Pleistocene population which inhabited a vast geographical area that extends from Portugal to western Siberia and from northern Europe to the Middle East. This population, according to the paleoanthropological data, descends from a European population, *Homo heidelbergensis* [1], [2], and the first Neanderthal features appeared at around 400,000 years BP [3], [4]. Neanderthals disappeared around 35,000 years BP with the arrival in Europe of *Homo sapiens* [*5*]–[*10*]. In such a vast area, paleoanthropological studies have reviewed variability among Neanderthals, and have identified different groups: one from Western Europe, one from the Middle East [11]–[14], and a third in the Southern region [15]–[18].

During the 1990s, research on Neanderthal genetics has developed with the emergence of the molecular biology. Since 1997 mtDNA of twelve fossils has been sequenced and these sequences constitute our sampling for this study [19]–[28]. Today, studies of Neanderthal genetics are focused essentially on the relationship between Neanderthals and modern humans and on their phylogenetic status [29], [30].

Our approach differs from earlier studies in that it considers the genetic variability only in Neanderthals. The objective is to understand the demographic structure and evolution of the Neanderthal geographic distribution by analyzing genetic variability and by modeling different scenarios. To this end, we made simulations, with the software BayesSSC [31], [32], which uses the coalescent method. These demographic and genetic simulations represent the potential demographic process affecting Neanderthals. They include priors such as the rate of mutation, the rate of migration among independently evolving sub-populations, population size and changes in it. At the end of each simulation, measures of genetic diversity were calculated. Next, we evaluated the adequacy of fit for each model. Following this we rejected several hypotheses regarding the geographic distribution and relationships among Neanderthals, and we confirmed some paleoanthropological assumptions.

## Results

In our methodology, we estimated four measures of genetic variation (observed measures of genetic diversity): number of haplotypes, h, haplotype diversity, Hd, nucleotide diversity, Pi, and pairwise difference, K with DNAsp software.

Then, we established several genetic and demographic models from non-nucleotidic data. These models differ by the number and the spread of geographical groups among the Neanderthal population. Simulated values of these measures of genetic diversity for these models (2000 values for each measure and for each simulation) were obtained by coalescence simulations with the BayesSSC software. Among the 16 simulations listed for each model, we tested different growth rates, different migration rates and a range of initial population sizes. Then, we compare Co (combination of the probabilities associated with observed values) with the C distribution (each of the 2000 C values is the combination of the probabilities associated with one set simulated measure of genetic diversity (h, Hd, Pi and K)), thus providing empirical posterior probability for the whole model. If the model corresponds exactly with observed genetic diversity, this empirical posterior probability should be equal to 0.5. Generally speaking, if we consider 12 or 9 sequences, all the models present plausible simulations, and this is particularly true when the population is growing and some migrations occur between demes, except for model 1 (table 1). However, we should not forget that when we work with 12 sequences, the length of these sequences is very small and weakly polymorphous (because it is a preserved sequence in the Neanderthal population). By contrast, with 8 or 7 sequences the results seem poorer; and we explain this by the few number of sequences considered. In other words, our best results are for simulations which take into account a sufficiently significant number of sequences (twelve or nine sequences). And in other cases (eight or seven sequences), lengths seem too short and simulated haplotype diversity is never in the range of the observed diversity.

**Table 1:** probability of the adequacy for each simulation (P(Co|C)) and associated P′ of four observed measures of genetic diversity.

Models	Number of sequences	Growth rate	Migration rate	Hap. number	Pairwise difference	Hapl. diversity	Nucl. diversity	P(Co|C)
Model 1	12	0	1	0.143	0.152	0.286	0.141	0.1240
		0	0	0.111	0.139	0.286	0.130	0.1205
		1	1	0.112	0.118	0.231	0.110	0.0905
		1	0	0.093	0.117	0.223	0.113	0.0830
	9	0	1	0.144	0.114	0.952	0.110	0.1305
		0	0	0.139	0.117	0.943	0.114	0.1235
		1	1	0.102	0.083	0.828	0.082	0.0875
		1	0	0.084	0.077	0.808	0.074	0.0860
	8	0	1	0.717	0.106	0.000	0.104	0.0030
		0	0	0.704	0.119	0.000	0.118	0.0025
		1	1	0.485	0.077	0.000	0.076	0.0010
		1	0	0.499	0.097	0.000	0.095	0.0025
	7	0	1	0.363	0.097	0.000	0.098	0.0025
		0	0	0.414	0.101	0.000	0.101	0.0010
		1	1	0.318	0.085	0.000	0.085	0.0005
		1	0	0.294	0.076	0.000	0.076	0.0025
Model 2	12	0	1	0.144	0.154	0.350	0.147	0.1470
		0	0	0.130	0.118	0.324	0.111	0.1140
		1	1	0.174	0.227	0.400	0.220	0.2025
		1	0	0.184	0.171	0.412	0.167	0.1790
	9	0	1	0.138	0.104	0.910	0.102	0.1210
		0	0	0.108	0.049	0.929	0.046	0.0695
		1	1	0.169	0.191	0.967	0.188	0.1895
		1	0	0.151	0.122	0.958	0.118	0.1375
	8	0	1	0.726	0.100	0.000	0.097	0.0005
		0	0	0.688	0.049	0.000	0.048	0.0020
		1	1	0.667	0.167	0.000	0.161	0.0005
		1	0	0.618	0.104	0.000	0.101	0.0015
	7	0	1	0.453	0.099	0.000	0.100	0.0015
		0	0	0.432	0.045	0.000	0.047	0.0010
		1	1	0.347	0.188	0.000	0.192	0.0050
		1	0	0.344	0.105	0.000	0.107	0.0050
Model 3a	12	0	1	0.146	0.141	0.272	0.138	0.1335
		0	0	0.058	0.048	0.195	0.045	0.0420
		1	1	0.226	0.293	0.445	0.285	0.2745
		1	0	0.170	0.143	0.404	0.138	0.1505
	9	0	1	0.108	0.075	0.966	0.073	0.0960
		0	0	0.075	0.024	0.915	0.022	0.0405
		1	1	0.164	0.227	0.959	0.222	0.2170
		1	0	0.136	0.098	0.929	0.094	0.1100
	8	0	1	0.595	0.057	0.000	0.053	0.0025
		0	0	0.601	0.026	0.000	0.026	0.0020
		1	1	0.603	0.220	0.000	0.214	0.0005
		1	0	0.597	0.078	0.000	0.076	0.0005
	7	0	1	0.391	0.063	0.000	0.064	0.0040
		0	0	0.361	0.027	0.000	0.027	0.0010
		1	1	0.346	0.171	0.000	0.173	0.0015
		1	0	0.314	0.058	0.000	0.058	0.0025
Model3b	12	0	1	0.119	0.116	0.251	0.110	0.1030
		0	0	0.099	0.074	0.254	0.067	0.0705
		1	1	0.210	0.275	0.424	0.268	0.2480
		1	0	0.160	0.154	0.399	0.141	0.1525
	9	0	1	0.093	0.069	0.920	0.064	0.0825
		0	0	0.092	0.041	0.860	0.040	0.0570
		1	1	0.173	0.221	0.898	0.217	0.2045
		1	0	0.139	0.108	0.865	0.105	0.1240
	8	0	1	0.578	0.062	0.000	0.060	0.0010
		0	0	0.546	0.021	0.000	0.020	0.0015
		1	1	0.528	0.226	0.000	0.224	0.0005
		1	0	0.470	0.069	0.000	0.068	0.0035
	7	0	1	0.339	0.066	0.000	0.066	0.0005
		0	0	0.302	0.021	0.000	0.021	0.0020
		1	1	0.271	0.168	0.000	0.169	0.0005
		1	0	0.275	0.075	0.000	0.076	0.0020
Model 3c	12	0	1	0.170	0.143	0.404	0.138	0.1505
		0	0	0.094	0.079	0.299	0.076	0.0760
		1	1	0.239	0.315	0.492	0.303	0.2900
		1	0	0.302	0.460	0.627	0.445	0.4190
	9	0	1	0.094	0.063	0.920	0.061	0.0840
		0	0	0.087	0.029	0.905	0.026	0.0410
		1	1	0.177	0.232	0.964	0.226	0.2190
		1	0	0.128	0.080	0.907	0.074	0.0935
	8	0	1	0.590	0.063	0.000	0.062	0.0015
		0	0	0.638	0.033	0.000	0.033	0.0020
		1	1	0.587	0.204	0.000	0.199	0.0005
		1	0	0.585	0.088	0.000	0.083	0.0015
	7	0	1	0.373	0.064	0.000	0.065	0.0040
		0	0	0.389	0.037	0.000	0.039	0.0010
		1	1	0.335	0.154	0.000	0.157	0.0010
		1	0	0.330	0.086	0.000	0.086	0.0025
Model 4	12	0	1	0.075	0.094	0.197	0.089	0.0710
		0	0	0.030	0.040	0.094	0.037	0.0165
		1	1	0.141	0.192	0.356	0.183	0.1690
		1	0	0.049	0.052	0.141	0.049	0.0370
	9	0	1	0.054	0.044	0.698	0.042	0.0490
		0	0	0.018	0.007	0.440	0.007	0.0115
		1	1	0.134	0.185	0.860	0.177	0.1730
		1	0	0.049	0.039	0.559	0.038	0.0450
	8	0	1	0.398	0.038	0.000	0.037	0.0030
		0	0	0.218	0.010	0.000	0.009	0.0025
		1	1	0.578	0.181	0.000	0.175	0.0005
		1	0	0.299	0.029	0.000	0.027	0.0020
	7	0	1	0.195	0.037	0.000	0.037	0.0010
		0	0	0.086	0.007	0.000	0.007	0.0015
		1	1	0.250	0.153	0.000	0.154	0.0030
		1	0	0.092	0.024	0.000	0.024	0.0020

Co = combined P-values for observed values.

C = distribution of combined P-values for simulated values 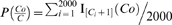.

growth rate ∶ 1 = 0.00016/0 = 0.

migration rate : 1 = 0.0002/0 = 0.

### Model 1: a unique population

This oversimplified model corresponds to an ancestral population which develops into a unique population. In this model two assumptions were tested: the first posited a constant population between 130,000 and 50,000 years BP; the second assumed a growing population over the same time period. We suggested a range of values for population size: {25,000–200,000 individuals} [33]–[35] (namely {3,125–25,000} for the effective population size) and a generation time of 20 years [36]. Sixteen simulations of this model have been tested. In all cases, the empirical posterior probability is far from 0.5, thus reflecting the inadequacy of this model in relation to observed data (table 1).

### Model 2: two derived populations

This model corresponds to an ancestral population which gives rise to two sub-populations: one in the West, the other in the East. This division reflects the paleoanthropological assumption of the morphological gradient from east to west. In this model we made two assumptions, first was constant population, second growing population. We further proposed two hypotheses regarding migration between the derived sub-populations: an absence of migration or a low migration between groups (with a migration rate of 0.02%). We also used a range of values for population size: {25,000–200,000 individuals} [33]–[35] (namely {3,125–25,000} for the effective population size). We assume that each sub-group had roughly the same population size, and we consider that the generation time was 20 years [36]. Sixteen simulations of this model have been tested. We observed that several simulations are plausible but that none present P(Co|C) values close to 0.5 (table 1).

### Model 3a/3b/3c: three derived populations

**Figure 1:**
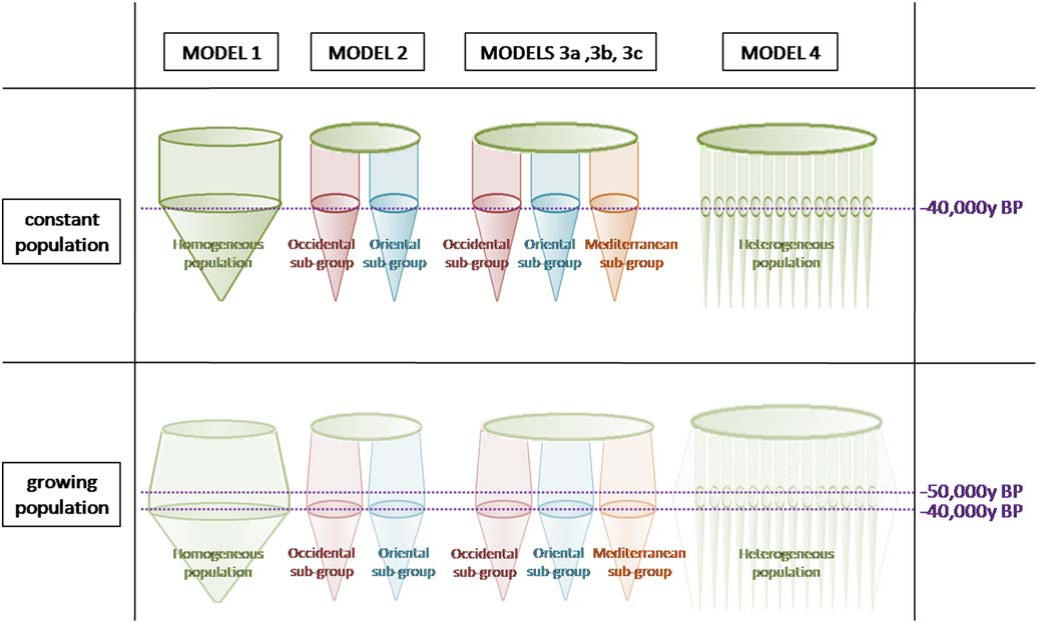
Schematic presentation of the six models.

The third models correspond to an ancestral population which gives rise to three sub-populations: one in the West, another in the East and one in the South (fig. 1, table 1). This southern population corresponds to the paleoanthropological hypothesis concerning the presence of a Southern population [3], [15]–[18]. According to the geographical barriers and morphological evidence, we have established three different divisions. The fossil of El Sidron from a paleogeographic standpoint is closer to French fossils than to Italian and Croatian fossils. On the basis of morphological data it might be closer to the southern fossils (model 3b). Due to its geographical position, the fossil of Mezmaïskaya, discovered in the Caucasus, might be placed either in the eastern (model 3a) or in the western group (model 3b and 3c). These divisions are shown in table 2. For model 3 (a, b, c) we made the same assumptions as in model 2 regarding population growth, migration, population sizes, and generation time. Forty eight simulations of this model have been tested, sixteen by grouping. Most measures of genetic diversity fit the observed measures more closely than in the previous models. Indeed, if we consider a growing population in which migration occurs, we see plausible and best values of P(Co|C) for all models (3a, 3b and 3c) for simulation sets with twelve or nine sequences. The most precise fit is that of model 3c, which presents values of P(Co|C) closest to 0.5 (table 1). If we consider a growing population with no migration, only model 3c presents the best values of P(Co|C). Thus models three, which posit three groups among Neanderthals, and assume a growing population, seem to be most realistic, and model 3c is the most plausible one.

**Table 2:** grouping supposed in each model.

*FOSSILS*	*Dates (years BP)*	*Seq. length*	*model 1*	*model 2*	*model 3a*	*model 3b*	*model 3c*	*model 4*
Teshik Tash (Ouzbekistan)	−45,000	191	group 1	*eastern*	*eastern*	*eastern*	*eastern*	group1
Okladnikov (Siberia/Russia)	−40,000	303	group 1	*eastern*	*eastern*	*eastern*	*eastern*	group2
Mezmaïskaya (Caucasus)	−29,000	303	group 1	*eastern*	*eastern*	western	western	group3
Feldhofer1 (Germany)	−42,000	303	group 1	western	western	western	western	group 4
Feldhofer2 (Germany)	−42,000	303	group 1	western	western	western	western	group 5
Engis2 (Belgium)	−35,000	31	group 1	western	western	western	western	group 6
Scladina (Belgium)	−100,000	111	group 1	western	western	western	western	group 7
La Chapelle-aux-Saints (France)	−45,000	31	group 1	western	western	western	western	group 8
Rochers de Villeneuve (France)	−45,200	31	group 1	western	western	western	western	group 9
El Sidron (Spain)	−43,000	303	group 1	western	western	**southern**	western	group 10
Monte Lessini (Italy)	−50,000	303	group 1	western	**southern**	**southern**	**southern**	group 11
Vindija 80 (Croatia)	−38,000	303	group 1	western	**southern**	**southern**	**southern**	group 12
Number of groups			1	2	3	3	3	12

### Model 4: a heterogeneous derived population

This other model corresponds to an ancient population which yields numerous sub-populations (12 sub-populations), represented by each available nucleotide sequence. Indeed, according to paleodemographical data on population density, we assume that each group was quite isolated and that exchanges between demes were certainly very low. In this model we made the same assumptions as in models 2 and 3 concerning growth, population size and generation time. For migration, we assumed a migration rate of 0.02% between adjacent groups, 0.01% between near groups and 0 between remote groups. Sixteen simulations of this model have been tested. In most cases, the empirical posterior probability is far from 0.5, thus it reflects the inadequacy of this model in relation to observed data (table 1).

## Discussion

Our study uses a new coalescent Bayesian method to generate information about human paleoanthroplogical populations. The methodology we have developed is similar to that employed in studies of *Belle et al.* [37], [38], and represents a novel way of analyzing the Neanderthal population structure.

This new method has allowed us to establish an ancestral sequence at around 130,000 years BP even if we lack available genetic data for this period.

In the framework of this short paper, we did have not provided a detailed examination of the Neanderthal population structure, but we have obtained a plausible idea of its general character, in particular concerning population growth and migration. Indeed, we found that the models with a growing population presented the highest overall probability. Nonetheless, models which do not take migration into account could not be rejected, but models which assume that migration occurred fit the observed data more adequately.

We have limited our study to what occurred previous to the arrival of modern humans in the Neanderthal landscape and we therefore do not consider the potential phylogenetic relationship between Neanderthals and modern Humans.

Since the number of available nucleotide sequences is very low, we had to make some assumptions and generalizations to approximate the genetic and demographic structure of Neanderthals. For example Scladina was the unique available sequence around 100,000 years BP and this could influence our results because the coalescence prossess based only on one reference for this time.

Sequence lengths are heterogeneous and quite short (31 to 369 pb); for this reason we had to do several sets of simulations, considering four different sequence lengths (31 pb/111 pb/191 pb/303 pb). Thus, we either have all the sequences, but short, or else few sequences, but long. There is no optimal case. Nonetheless, all the sequences contain the characteristic sequence of Neanderthals. In addition, the use of sequences from the HVR1 region of mtDNA has permitted us to consider nucleotide variations. Indeed in order to employ the coalescent method, we had to analyze a quite variable sequence with as many polymorphisms as possible. The recent sequencing of other parts of the mitochondrial genome [39] could provide new information and will help us refine our models in the future.

The models we established according to the paleoanthropological data enabled us to represent only the potential structure of the spread of the Neanderthal population. The results of our study support certain paleontological scenarios. The results of statistical analysis indicate that models 1, 2 and 4 are less plausible than models 3.

Models three and particularly model 3c are also statistically the strongest models. Moreover, they are coherent with the paleoanthropological data. Neanderthal features favour a distinction between western and eastern Neanderthals, with the western populations presenting more derived features than the eastern groups [13], [14]. This distinction has been explained by a migration to the East during the isotopic stages 5 or 6 [12], [14] or by a phenomenon like isolation through distance [40]. The sequence of Mezmaïskaya (in the Caucasus) is thus located in an intermediate region and could be placed in either of these groups. According to the geographical position of this fossil, this sequence could also belong to still another group. In the state of our knowledge, however, we are unable to test this assumption, since we need at least two sequences to establish a group. These models, 3a, 3b and 3c, also support previous paleoanthropological studies of the phylogenetic position of the fossil remains of Okladnikov in Siberia [26]. This fossil has been identified as a Neanderthal on the basis of its nucleotide sequence. Our results situate this fossil in the eastern Neanderthal group.

Studies of the skull [3], [15], [17], teeth [15], [18] or upper limbs [16] have suggested a possible third group, the southern group, but its existence is subject to debate among paleoanthropologists. The geographical extension of the southern group varies according to the authors. For some of them, all of the fossils of the Iberian peninsula belong to the southern group, for others the fossils from northern Spain belong to a group extending from Spain to Belgium and these authors limit the southern group to the eastern coast of the Iberian peninsula, the south of France, the Italian peninsula and the western part of the Balkans. These assumptions are explained by climatic conditions particular to the southern region and by the eco-geographical barriers which could have had an impact on the mobility and spread of Neanderthals. Our results clearly favour the existence of one southern group which is present throughout model three. Our models 3b and 3c differ in regard to the position of El Sidron (northern Spain). According the model 3b the fossil of El Sidron is located in the southern group, wheras the model 3c situates it in the group extending from Spain to Belgium. In addition, we supposed that sequences of Monte Lessini in Italy and Vindija in Croatia allow to the same group because it exists a morphological closeness. This can be explained in terms of eco-geographical factors. Indeed these two regions are isolated in the north by the Alps and, in the range of datings that we consider for these sequences, a marine regression offers an easier passage between northern Italy and Croatia.

The absence of sequenced fossils in the Near East does not permit us to situate these fossils in any of these groups. This is unfortunate, since their geographic position might have permitted us to situate them in the southern group or in the eastern group, or even in their own group, a fourth one, in which the sequence of Mezmaïskaya could be placed.

**Figure 2:**
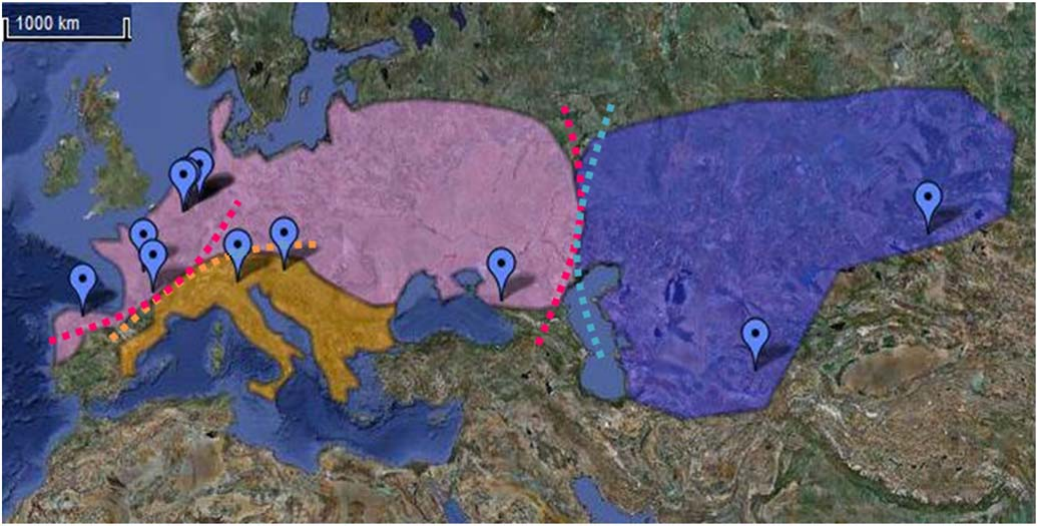
Map representing Neanderthal geographical distribution in groups.

In conclusion, our approach to Neanderthal variability, based on nucleotide sequences analysis, confirms from a genetic point of view the morphological variations between western and eastern Neanderthals and the existence of a southern group (fig. 2). Moreover, it seems from our results that the Neanderthal population was not constant over time and that some migration occurred between the demes.

## Materials and Methods

### mtDNA sequences

This study focused on twelve sequences of the hypervariable region I of mtDNA. These sequences represent the twelve Neanderthal fossils which have been sequenced since 1997 [19]–[28]. These fossils have been dated between 100,000 and 29,000 years BP and are located in an area extending from Spain to Siberia corresponding to the major part of the Neanderthal territory. Unfortunately, we have only one fossil dated approximately 100,000 years BP and there is a gap between 100,000 and 50,000 years BP. These sequences were grouped together and could thus represent several sub-populations (table 2).

### Software used

First we used DNAsp software to extract a genetic differentiation parameters set from nucleotide sequences (haplotype number, haplotype diversity, nucleotide diversity and pairwise difference). Next, for modelling the evolution of the structure of the Neanderthal population, we used Bayes SSC (Bayesian Serial SimCoal) software [31], [32]. This software uses a coalescent method. A bayesian approach is employed to test demographical scenarios from ancient DNA data and to get approximations of the likelihood of one scenario in relation to another. We reviewed many demographic and genetic scenarios. For each scenario, the software employed several priors: the number of groups (or demes), the dates of each sequence/fossil, the membership of different groups, the population size at the beginning and at the end of coalescence, the growth rates of sub-populations, the migration rates, the mutation rates, the heterogeneity rate, the transition/transversion bias and the length of sequences that we sought to simulate. For each scenario, we simulated 2,000 genealogies and we obtained a set of genetic differentiation parameters (the four measures of genetic diversity), the same as those obtained by DNAsp. For comparing data generated by Bayes Serial SimCoal with data extracted directly from mtDNA sequences, we performed statistical test proposed by Voight et al. 2005 [41].

### Models of Neanderthal population structure

In this study, we reviewed six basic population models, which were complemented by a considerable number of genetic and demographic priors (fig. 1). In the six basic models, each nucleotide sequence represents one part of the population (table 2). These models differ by the number and the composition of sub-groups among Neanderthals. Indeed, the distribution area of Neanderthals is widespread and we might wonder if the population in this geographic area was homogeneous or divided into groups. In order to answer this question, we established several models, one corresponding to a homogeneous population and the others to a population divided into different groups. The composition of the groups for our simulations is based on paleoanthroplogical assumptions derived from eco-geographical and morphological data. In each model we proposed several values for each prior: a range of values for the population size, two values for the growth rate, several values for the migration rate, a range of values for the mutation rate, one value for the heterogeneity rate and four lengths of the nucleotide sequences. The mix of these prior values yielded numerous simulations. Nevertheless, in all the simulations the ancestral population, at around 130,000 years BP, is constant or growing; and it then begins to decrease at around 40,000 years BP until its full extinction at approximately 25,000 years.

### Demographic priors

The first demographic prior used is population size. Since very little data is available concerning the population size of the Neanderthals, we relied on data from Upper Paleolithic *Homo sapiens*. For this we applied two extreme estimates: 200,000 individuals as suggested by Biraben [34], and 25,000 individuals, as extrapolated from data from the Upper Paleolithic [33]–[35]. Because the effective population size for mitochondria is approximately one fourth of the autosomal population size, and taking effective size to be approximately one half of census size [37], [42], [43], the effective population size is approximately one eighth of the census size namely: 25,000 and 3,125 number of copies of the gene. Thus, we proposed a range of {3,125–25,000}.

The second demographic prior is the growth rate. This is an exponential rate for which we have proposed two assumptions: 0 and 0.00016 which have been extrapolated from Upper Paleolithic data [33]–[35]. Next, we considered a decrease in the Neanderthal population following the arrival of *Homo sapiens* in their territory. The exponential rate we calculated is −0.0064. We realise that the short decrease time (15,000 years) attributed to the Neanderthal population introduces a bias, since we don't consider other factors such as climatic or environmental parameters which might also have had an impact on the weakening of population growth.

The third demographic prior is the migration rate. Here we proposed two assumptions: the first concerns an absence of migration, the second a low migration rate of 0.0002 [44] between each adjacent group, 0.0001 or 0 between more distant groups. These values may seem low but they are proportional to the range of population size we have chosen, and a larger value would therefore not be appropriate.

### Genetic priors

The mutation rate is the first genetic prior we considered. Because this rate is very difficult to determine, and even more so for Neanderthals, we have propose a range of possibilities: {5%–125%} of difference of sequence by million years [36].

The second prior is the heterogeneity rate. For this we chose the classical rate of 0.26 [36].

For the third prior, we considered the sequence length. The twelve mtDNA Neanderthal sequence lengths are heterogeneous (31 to 369 pb) and, for this reason, we had to do four sets of simulations (with 12 sequences, 9 sequences, 8 sequences or 7 sequences), considering four different sequence lengths (31 pb/111 pb/191 pb/303 pb).

For the transition/transversion bias, the fourth prior, we chose to take a bias directly calculated on the basis of nucleotide sequences by the software MEGA4 [45] (61.432 for twelve sequences/254.911 for nine sequences/9.405 for eight sequences/15.683 for seven sequences; this value is calculated for a heterogeneity rate of 0.26).

### Comparison of observed and simulated data

For each simulation, we calculated several statistics for four measures of genetic diversity (total number of haplotypes, haplotype diversity or heterozygosity, average pairwise difference and nucleotide diversity). To obtain a measure of the adequacy of fit for each model, we adopted the criteria described by Voight et al. [41]. We used coalescent simulations to generate 2,000 genealogies for each set of parameter values. We compare each simulated measure of genetic diversity, x_n_, with the other 1999 values. The probability, *P*, is the probability of observing, in the distribution of simulated measures of genetic diversity, values greater than each considered simulated value, x_n_. This probability is converted to a two-tailed *P′* value by applying the formula 1−2*|0.5−*P*|. Then, we calculated the statistic C which combines the *P′* of four measures of genetic diversity (haplotype number, haplotype diversity, pairwise difference and nucleotide diversity) as follows: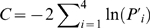 We repeat this on the 2000 simulated measures to obtain a null distribution of C. We proceeded in the same way with the observed measure of genetic diversity to obtain an observed C value called Co. Finally, we compare Co with C distribution, thus obtaining empirical posterior probability for the whole model (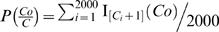).
